# Microbiome yarns: The Global Phenotype‐Genotype Survey. Episode III: importance of microbiota diversification for microbiome function and biome health

**DOI:** 10.1111/1751-7915.13406

**Published:** 2019-04-04

**Authors:** Kenneth Timmis, Franziska Jebok, Manfred Rohde, Leo Lahti, Gabriella Molinari

**Affiliations:** ^1^ Institute of Microbiology Technical University Braunschweig Germany; ^2^ Institute for Educational Science University of Freiburg Freiburg Germany; ^3^ Central Facility for Microscopy Helmholtz Centre for Infection Research Braunschweig Germany; ^4^ Department of Mathematics and Statistics University of Turku Turku Finland

BBZ, Studio 7A, BBZ Plaza, Burbank, 7.30 pm^1^^,^ 2 ^,^ 3 ^,^ 4

*Abigail Repor‐Tastory,* 5 *Discovery Presenter, turns to face the camera*:

Good evening viewers and welcome to a new episode of ‘Discoveries that Change our Lives'. Our guest this evening is once again Dr. Anastasia Noitall‐Most^5^ from the Streber Elite University of Los Angeles.^5^ Good evening Dr. Noitall‐Most *(shaking hands)* and thank you for appearing on the program.

*Dr. Noitall‐Most:* Good evening Abi; it is always a pleasure to be here.

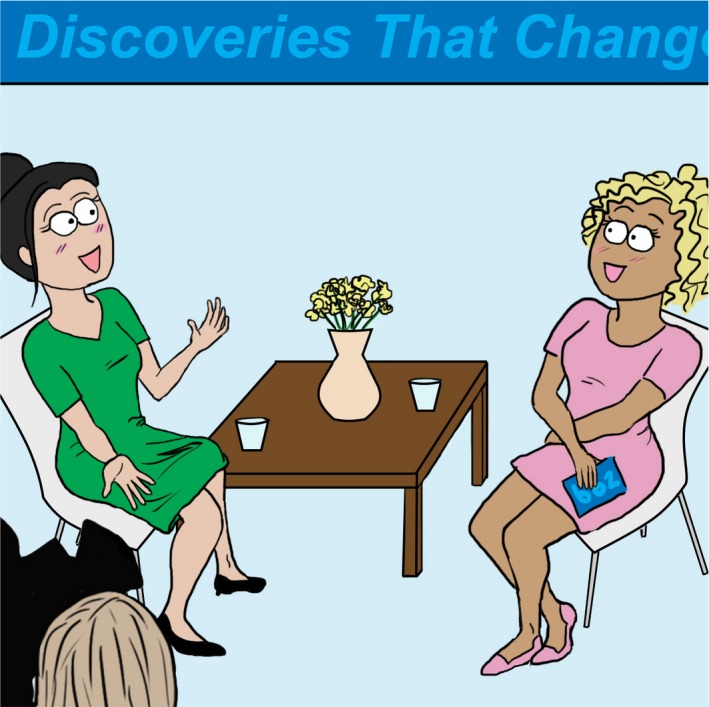

*Ms. Repor‐Tastory*: Ani, this evening we will continue the discussion we began in the last episode about the results of the most ambitious human microbiome survey to date, the Global Phenotype‐Genotype Survey, or *GLOPS*.^6^

*Dr. Noitall‐Most:* Yes, Abi: *GLOPS* taught us that each human being has a unique genotype‐phenotype, determined by its integrated human and microbiome metagenome, but that certain human:human behavioural activities lead to the sharing of some of the microbiota and a resulting short term or, more rarely, longer term changes in metagenotype‐phenotypes, including voice quality and diction.^6^

This evening, I'd like to explore some fascinating findings of *GLOPS* concerning non‐human, *environmental* mediators of what we might call microbiome diversification and diversity maintenance. Of course, most people by now have heard that subways are environments where spectacular microbiota exchanges can take place, in the case of the Hong Kong subway, where cross border exchanges, including the sharing of antibiotic resistant bugs and genes, are effected.^7^

*Ms. Repor‐Tastory*: Gosh, yes, that was a bombshell at the time!

*Dr. Noitall‐Most:* Indeed! And of course there are other activities, outside of subways, that do the job, including money handling.^7^^,^ 8 And, of course, *GLOPS* provided ample confirmation of the widely‐held belief that domestic animals, particularly pet dogs, significantly boost intestinal microbiota diversity.^8^

*Ms. Repor‐Tastory*: Yes, this led to an amazing increase in the sale of pets, especially dogs, in families with young children: it was impossible to keep up with demand in the months following this finding, despite an extortionate increase in prices! And the stocks of manufacturers of pet food and accessories became the favorites of the tipsters and went through the roof!

*Dr. Noitall‐Most:* Yes, unfortunately, I missed that train when it started to roll. But, a totally unexpected finding by *GLOPS* and its subsequent causality trials is that it is not only *changes* in our microbiota composition that affect us. It has now become clear that new microbes that pass through/over us, but *fail* to set up home in our resident microbiome, can have significant consequences for us. In other words, microbial *transients* also play an important role in our health and behavior.

*Ms. Repor‐Tastory*: But if they don't take up residence, how can they possibly affect us?

*Dr. Noitall‐Most:* Yes, that is a surprise and I will come to the likely explanation shortly. What is clear is that regular exposure to a richly diverse external microbial flora can have a major positive influence on our health and robustness. In terms of microbiome enrichment from the environment, the majority of us who do not have pet dogs that devotedly lick our faces all the time acquire new microbes principally by eating/drinking and breathing. And, since much of the food we eat is cooked, and drinks tend to have low numbers of microbes, our main source of microbial augmentation is in the air we take in by nose and mouth. Of course, the particulate content of air ‐ solids like dust and water droplets/aerosols – and the microbiota they transmit, as well as individual microbes on a float‐about, vary enormously in time and place, with high levels experienced for example in the path of a dust storm, downwind of a composting plant, during a fog, etc.^9^ Similarly, the nature and diversity of air microbiota vary considerably according to location and wind direction, as do the beneficial effects on our health.

*Ms. Repor‐Tastory*: Gosh, yes: taking the air along the edge of a rapeseed field in full bloom in early Summer, with the nasal passages succumbing to a spirited pollen assault, and the air thick with insects, gives the feeling of breathing lentil soup!

*Dr. Noitall‐Most:* Yes, and of course pollen is a major component of the non‐nitrogen, non‐oxygen part of air that we inhale during the tree reproductive season and, at specific times in the farming calendar, like the one you mention. Interestingly, pollens have their own specific microbiomes,^10^ so act as carriers for microbial enrichment of our airways and GI tract. However, the diversities of pollen microbiomes are not enormously high and their beneficial effects on stimulating the immune system are somewhat counteracted by the histamine storm that pollen provokes in many people, when they venture outside. And, interestingly, the pollen microbiome has also been implicated in pollen allergy.^10^

In any case, the newly‐appreciated beneficial effects on our health of microbially‐laden aerosols has led to unexpected and explosive price increases in city houses near waste treatment plants, municipal composting facilities, well‐tended leafy parks subjected to a weekly blower coiffeur that collects vegetable and other matter into nice small piles and creates a miasma of microbially‐laden dust for locals to respire and ingest, airport runways and helicopter pads, and in country properties downwind of domestic animal residences, like dogs' homes and horse stables, farm husbandry buildings, including poultry houses, farm animal markets, etc., that are regularly *mucked out*, and near mixed farming land where pig slurry is periodically sprayed on the fields.^9^ Long gone are the days when extortionate house prices were justified by the poshness and aristocratic credentials of neighbours, and the distance to well‐regarded and highly performing schools!

*Ms. Repor‐Tastory, wrinkling her nose disapprovingly*: Yes, this trend is now the major topic of conversation at cocktail parties, but the prevailing odour after the slurry cart has been out and about can be overwhelming and promptly drives inside everyone enjoying cocktails on the terrace.

*Dr. Noitall‐Most:* Of course but, with time, our senses get accustomed to such stimulations and relegate them into our perception background. And, dare I say it, we then start to appreciate such smells as key ingredients of *good country air*!^11^

Anyway, at this juncture, I might also mention that it has recently been shown that a large fraction of microbes in the gut microbiota are spore‐formers, 12

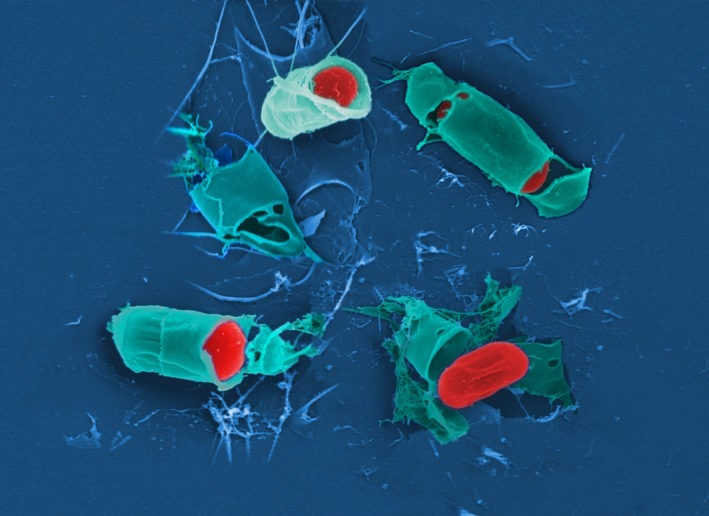

and other stress‐resistant forms, and an important conclusion drawn from this finding is that it is such forms that are preferentially shared between people.^12^ Of course, much of the environmental microbial bounty that is blown around in the dust, which we ultimately take into our airways and gut systems, also consists of spores and resistant forms, so it seems likely that it is spore‐formers which are primarily responsible for environmental boosts to our microbiota diversity.

*Ms. Repor‐Tastory:* Hhmm: I must confess that I hold my breath when passing someone creating a dust storm with one of those leaf blowers!

*Dr. Noitall‐Most:* Yes, Abi: this is a normal reaction ‐ the stuff they suspend in the air is not particularly appetizing, given that it contains all sorts of animal gifts and, anyway, one can always have too much of a good thing.

On the other hand, as is well known, we humans do not make all the vitamins and other metabolites we absolutely need for good health, nor are they present in adequate quantities in the food we consume.^13^

It has always been assumed that our gut microbes supply us with all the essential nutrients we do not make ourselves, in exchange for the privilege of the nice warm and cosy, food‐rich accommodation we provide. However, there is an increasing indication that some of our microbial supply of vitamins and other micronutrients does not come from microbial synthesis and release on site, *in entero* so to speak, but rather from the lysis of microbes in the gut,^13^ resulting from our own digestive processes and from the unfriendly activities of predatory bacteria and microbial viruses.

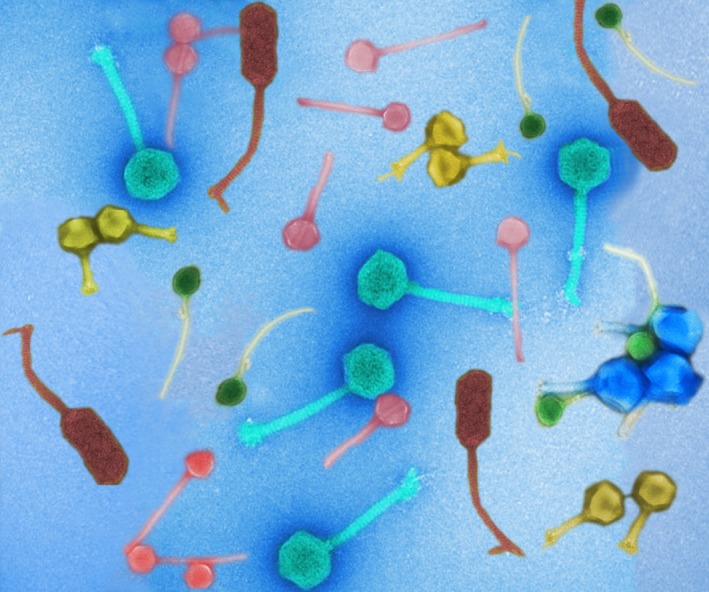

A good example of this is vitamin B12, which is not made by plants and animals and only by a limited number of microbes, and apparently not released in sufficient quantities by our growing gut microbes to satisfy our needs. So the question is: how do we acquire enough? Interestingly, carnivorous predators, especially hyaenas, vultures, etc., often prefer the delights of the entrails of the prey, rather than its juicy meat, and many animals, like rodents, rabbits, dogs and even primates and, to some extent, piglets and foals, practice coprophagy – the eating of faeces. According to a world‐renowned expert,^4^^,^ 13 the microbial contents of the GI tract may be an important ‐ hitherto unappreciated ‐ supply chain of essential trace nutrients present in microbes that we digest and thus exploit as food.

*Ms. Repor‐Tastory:* Coprophagy? Uuhhh: how disgusting! …………………..Primates, you say?

*Dr. Noitall‐Most:* Yes, but obviously in the case of human primates, recognition of the importance of the oral‐faecal route for transmission of infectious diseases has somewhat dampened our enthusiasm for coprophagy – in fact we have become coprophobic, and developed other supply chains over time. One may be a high meat diet; another may be co‐evolution of humans and our microbiota, resulting in our microbiota containing sufficient numbers of those bugs able to produce B12 which, after careful cultivation, we sacrifice to extract their vitamin content. And a third may be a regular intake of a goodly quantity of microbial bounty from the environment. So perhaps when you pass a nice fella wielding a leaf blower, instead of holding your breath, you might consider the possibility that his efforts to herd leaves may concomitantly be unwittingly herding vitamin‐rich microbes in your direction to supplement your diet, and which may reduce your need to continuously pop vitamin pills.

*Ms. Repor‐Tastory:* Gosh: that is interesting! I spend a small fortune on vitamin supplements which otherwise could be used to upgrade my gym subscription.

*Dr. Noitall‐Most:* Exactly, but let's move from microbes as food supplements back to microbes as agents of human microbiota diversity. In addition to the aforementioned sources, there are other provenances of transient microbe immune stimulation. Most of us, when we exit from the built environment, breathe in significant quantities of frass particles – insect excreta – with heavier doses delivered in certain settings, like near beehives, wasps nests, etc., which provide a steady stimulation of the lung immune system, as well as sometimes contributing to the development of asthma.^14^ And then there are those individuals who regularly program the uptake of significant quantities of diverse microbes, like farmers, butchers, fisher(wo)men, cleaners, etc., who handle microbially‐rich materials, and who may unconsciously sample the rich flora of their unwashed hands during mealtimes. And sword swallowers. And then there is the special case of gardeners, who joyfully eat the bounty of their efforts plucked directly from the soil or tree, or who bite their nails or suck their thumbs, and thereby enrich their microbiota with new friends from soil, plant surfaces, manure, and anything else they happen to handle. Just think of the rich menagerie of worms and insects, and their intestinal microbiota, in a nice compost heap. And then there are plumbers….

*Ms. Repor‐Tastory:* Oh absolutely! Most of the gardeners and plumbers I know are rather earthy folk, but all the nicer (and healthier) for it. I vividly remember the explosive sneezing attack I experienced after diving into a haystack with a cute, rosy‐cheeked farm boy one harvest time……..*……(angry noises emanating from the in‐ear headphone)*…Oh, sorry, I did not mean to interrupt…

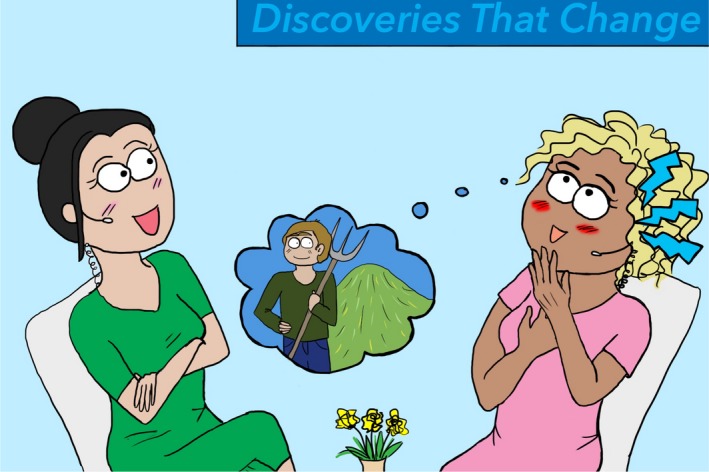

*Dr. Noitall‐Most:* Yes: a sneezing attack can be distracting, even downright awkward, in some situations.

*Ms. Repor‐Tastory:* But anyway, why does exposure to lots of different bugs that *fail* to incorporate in our microbiome have such a positive effect?

*Dr. Noitall‐Most:* Well, we do not yet have definitive information on this score but, according to the *GLOPS* consultant, Professor Tim Kennis of the Queenton Institute of Advanced Studies, a primary duty of our mucosal immune defenses is a sort of guard duty: to maintain surveillance of our mucosal surfaces, in order to recognize the arrival of pathogens and their establishment in our microbiota, and to promptly attack them to ward off an imminent infection. However, when the microbiota composition does not change much, the duty guards relax, get bored and become lazy. Current lifestyles, which are increasingly dominated by work‐at‐home flexi‐employment, entertainment consisting more of computer games, streamed music and movies, rather than of microbe‐enriching activities like soccer, rugby, cross‐country running, etc., and remote social interactions by social media, home delivery of all essentials, and only infrequent surreptitious sorties from the home to replenish cigarette supplies or visit the local school on parent‐teacher days, have enormously reduced our exposure to new bugs and are leading to progressive atrophy of our immune systems, a good example of the maxim *if it is not used it withers*. This is consistent with the fact that the severity of typical infections regularly experienced by chair‐bound people, like colds and flu, seems to be increasing, not only because of pathogen evolution to greater virulence^15^ but also, Kennis moots, because our immune systems mount slower and weaker defense responses.

*Ms. Repor‐Tastory:* Oh: that would explain why Winter colds seem to hang on longer and longer each year, sometimes one bronchitis merging with the next cold, as if the bugs were treating us like a baton, always to be handed over to the next!

*Dr. Noitall‐Most:* On the other hand, continual exposure to new bugs results in regular microbial altercations on epithelial barrier surfaces, involving exploitation of physical, chemical and informational weaponry, as the new bugs try to displace resident bugs and gain traction, and in a marked perturbation in the microbe:mucosa communication traffic. All of these mucosal battles keep the immune system awake and primed, and prevents it from atrophying, hence the health benefits of lifestyles and situations that continually expose us to high doses of diverse bugs. Of course, having good immune defenses results in milder infection episodes, reduced development of co‐morbidities, reduced need for antibiotic therapy, and hence a reduced burden of antibiotic resistant bugs in the microbiomes of such people. An exception to this generalization is the acquisition of *pavement bounty* – the rich diversity of bugs on pavements – by individuals who get pickled^16^ during Saturday night clubbing, after which they are amazed to discover themselves lying on the pavement with their tongues out until rescued by a good Samaritan, because pickling also damps down immune responses, quite apart from other negative impacts on health, and hence negates any benefits of the rich microbial intake.

*Ms. Repor‐Tastory, screwing up her fact contritely:* Yes, this only happened to me once and was soooo embarrassing that I vowed never to indulge like that again, at least outside the privacy of my own home. But you are quite correct: the world, as viewed from the floor looks very different!

*Dr. Noitall‐Most:* Ah: you bring up an important point! When we consider microbial intake via air, we implicitly assume air at a level of about 1.6m, the average nose‐mouth height of adults…… a bit more for the Dutch, Montenegrins and Scandinavians.^16^ However, in addition to the occasional character who gets pickled, all infants spend considerable periods of their lives with their noses‐mouths close to the floor. Moreover, their joyful exploratory scrabblings in the minimal gravity impact zone resuspend the dust that had previously settled and quietly accumulated on the *to‐be‐explored* floor,^16^ so they actually create their own air composition to vacuum up, which is very different from the air Mum and Dad breathe in, 1.6m further up.

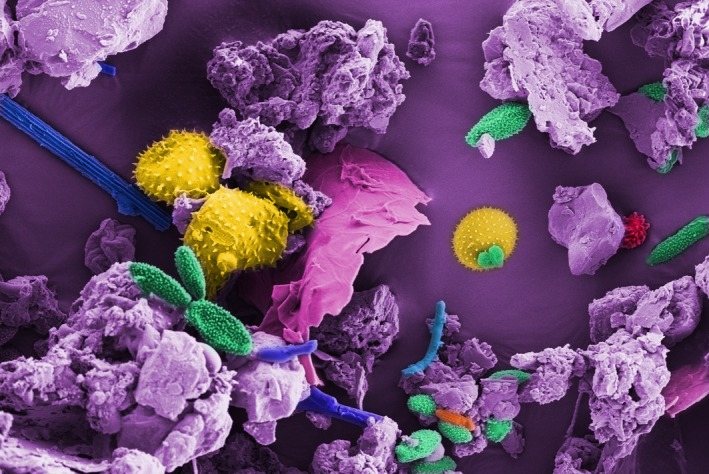

Dust^17^

*Ms. Repor‐Tastory, with a thoughtful expression on her face:* Yes, and it is not only the dust on the floor: all the stuff we bring in on our shoes, drop/spill on the floor, the dog's hairs; oh my goodness, and what about when Fido get an itchy bottom and drags himself along the floor on his rear end!

*Dr. Noitall‐Most:* Erm…yes: and this is only what they vacuum up indoors; just imagine what they sample outside, where the title of the book: *Let them eat dirt* comes into operation^18^! I think we can safely conclude that children have effective innate mechanisms to acquire a goodly diversity of microbes that will steer development of their microbiota and immune systems. Unless these mechanisms are frustrated by well‐meaning mums who continually strive for a sterile, pristine home environment.^18^

Anyway: to return to the usual situation, 1.6m up, where the microbe‐laden air delivers its microbial diversity bounty to our airways and GI tract.

*Ms. Repor‐Tastory, looking vaguely doubtful:* Yes, well, I suppose having cocktails on the terrace while surveilling the approaching air sparger‐generated haze of microbially‐saturated vapour wafting over from the waste treatment plant, and surrounded by the hunt pack going crazy, racing between people's legs, might have its compensations…..But anyway, didn't I read somewhere that compost heaps and the like contain pathogenic bugs?

*Dr. Noitall‐Most:* Aahh, I'm very glad you raised this issue, Abi! In fact, there is a plethora of publications attesting to the presence in all sorts of environments, including shower curtains, of microbes which belong to species which contain representatives that sometimes cause infections.^19^ However, such so‐called pathogens mostly cause disease in patients that are hospitalised and often have compromised immune responses – the so‐called *nosocomial* or hospital‐acquired infections.^19^ In fact, with a few exceptions, there is little robust epidemiological evidence for the pathogenic ability of most environmental bugs related in some way to microbes causing nosocomial infections, and hence for them to represent a health hazard. Moreover, phylogenetic assignments regularly change as they are updated, entraining alterations in previously‐proposed (perceived) relationships with other bugs, so assigning a taxonomic label that relates an environmental bug to a microbe that has at some point in the past been implicated in an infection, and then concluding without any causal evidence that the environmental relative is a hazard, is a highly dubious practice often used in an attempt to add attention‐catching drama to descriptive studies lacking design to test the hypothesis that is proposed. Even bugs with pathogenic potential are often *opportunistic* pathogens,^19^ that are mostly harmless for healthy individuals, and only possess *potential* to cause infections, mostly in immunocompromised hosts. Moreover, such bugs in the environmental samples are almost always only detected by metagenomic sequencing, almost never isolated and, where they are, almost never tested for their ability to cause disease in some standard infection model or other. Typical publications, mostly in the *Journal of Terribly Important Studies*,^20^ involve a few metagenome sequences and some handwaving about potential dangers, with a conclusion that the findings merit further investigation. Having done the easy bit and published a paper, the authors move on sharply, leaving the difficult bit of establishing causality to others, which usually means it never gets done: so‐called *throw over the fence‐*type research.

*Ms. Repor‐Tastory:* Well, that is most unsporting!

*Dr. Noitall‐Most:* Absolutely! *And*: most studies are only qualitative: a bug is shown to be present in the sample. But as all microbiologists know, an infection requires an *infective dose*,^21^ that is: a pathogen is able to cause disease only when present in sufficient numbers. Infective doses are pathogen‐specific: for example, among the diarrhoea‐causing bacteria, an infective dose of *Shigella* may be a single bacterium, whereas an infective dose of *Vibrio cholera* may be as high as ten million. And, switching from an environmental lifestyle to a pathogenic lifestyle is very resource costly,^22^ involving a major genetic expression program switch and the expenditure of much energy to produce all the microbial weaponry needed for the ecological battle with the host that is the basis of an infection. As a result, pathogens do a body count – the so‐called quorum sensing process^22^ ‐ to assess whether the effort will be worthwhile, before committing to the *Jekyll and Hyde* 23 harmless‐to‐harmful change. Practically no‐one who discovers potential pathogens in environmental samples takes the trouble to estimate, or even approximate, the numbers present in the sample and, more importantly, the potential doses likely to reach individuals at risk, and whether or not these might constitute infective doses.

In fact, public health agencies have pretty much defined real sources and routes of environmental infections, like water supply lines and cooling systems for things like *Legionella*,^24^ shellfish for *Vibrio*,^24^ freshwater bodies for *Leptospira*,^24^ etc. But most of the things we really need to worry about mostly come directly from other humans, either through physical contact, aerosols produced by coughing‐sneezing, or via food or water contaminated by faecal material.

*Ms. Repor‐Tastory:* Oh, well, that is a relief!

*Dr. Noitall‐Most: And*, to put all of this into context, our own microbiome contains these types of bugs, both potential and real pathogens, and we live with them in perfect harmony most of the time. I wonder how many authors of papers exposing the dangers of compost heaps and the like are aware of the bugs with pathogen potential on and in their own bodies, and those of their children and pet cats and dogs, what conclusions they would draw from such information and, importantly, what concrete measures they would undertake in response?

*Ms. Repor‐Tastory*: Well, yes, but I must admit to a certain aversion to dribbling babies and slobbery dogs that have just returned from walkies.

*Dr. Noitall‐Most:* Talking about dogs, I must share with viewers new information about how they distribute their microbial bounty to us. A fascinating aspect of the influence of dogs as family pets that was explored by the renowned mathematician, Professor Fidget Jones,^25^ who served as the statistician, modeller and Deputy Coordinator of *GLOPS*, was the importance of *slobber*, you know, the frothy stuff decorating and dripping from the mouths of excited dogs that is periodically centrifugally jettisoned by vigorous flicks of the head, especially when a flea in the ear decides to go on a walkabout to make sure her canine friend is awake and paying attention. The force and trajectory of which may be modulated by Fido's tongue. Well, it turns out that slobber is a perfect growth medium for many microbes that end up in the mouth, especially the vast array acquired during daily walkie sampling campaigns, so is chock‐a‐block with an amazing diversity of bugs. Anyone within range of the head flick shower is immediately gifted substantial numbers of all sorts of exotic microbes originating from the varied local flora and fauna inhabiting or visiting the walkie routes.

*Ms. Repor‐Tastory, indicating intense distaste:* Uuhhh! You mean that I need to be sprayed by that disgusting stuff in order to keep my immune system alert?

*Dr. Noitall‐Most:* Sorry Abi: at least two books by famous microbiomologists carefully document the issue of why dirt is good for us.^18^ But to return to the topic of discussion: in addition to its occasional violent ejections, slobber is also distributed more discretely though dripping and licking. Of course, the amount of slobber produced and shared with us does vary quite a lot from dog to dog, which raised the issue of interpretation and predictability of slobber production. Therefore, Fidget decided to formulate an equation that would provide a *slobber index*, or *SLIND*,^26^ which specifies the degree to which family members are regularly gifted, via their canine family member, with a microbial bounty.

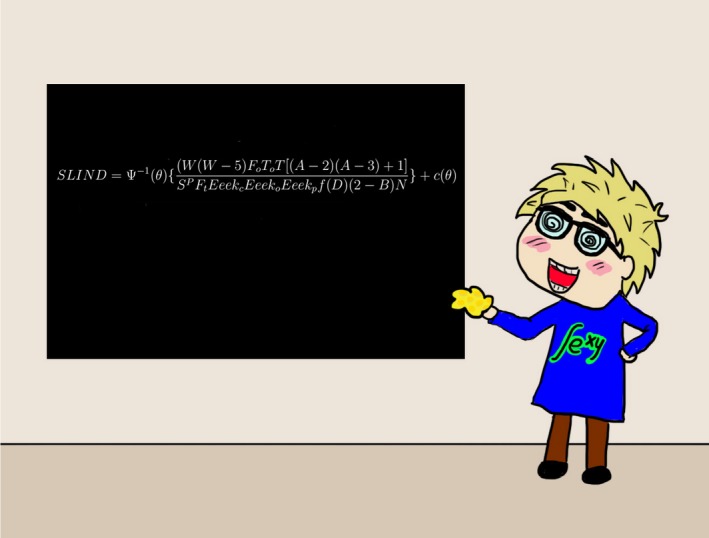

From a *SLIND* value, Fidget is able to accurately predict the diversity, the so‐called *α diversity*, of skin microbiota and, with slightly less precision, that of the gut microbiota, of dog‐owning individuals. The *α diversity* value is then employed as a key parameter in a new allergy susceptibility model which generates personalized predictive allergy susceptibility indices.

*Ms. Repor‐Tastory:* Well, I never: so slobber production can be an important contribution to the health of our microbiota and may influence our susceptibility to hay fever in the Spring!

*Dr. Noitall‐Most:* Yes, and it was this that induced the Imaging Group of Mabriella Golinari and Ranfredy Mohde of the Walpur Gisnacht Institute for Cellular Pathology in Bad Hurzbarg in Northern Germany,^27^ which was responsible for microbe isolation and characterisation in the *GLOPS* study, to explore the possibility that slobber production was influenced by buccal microbes.

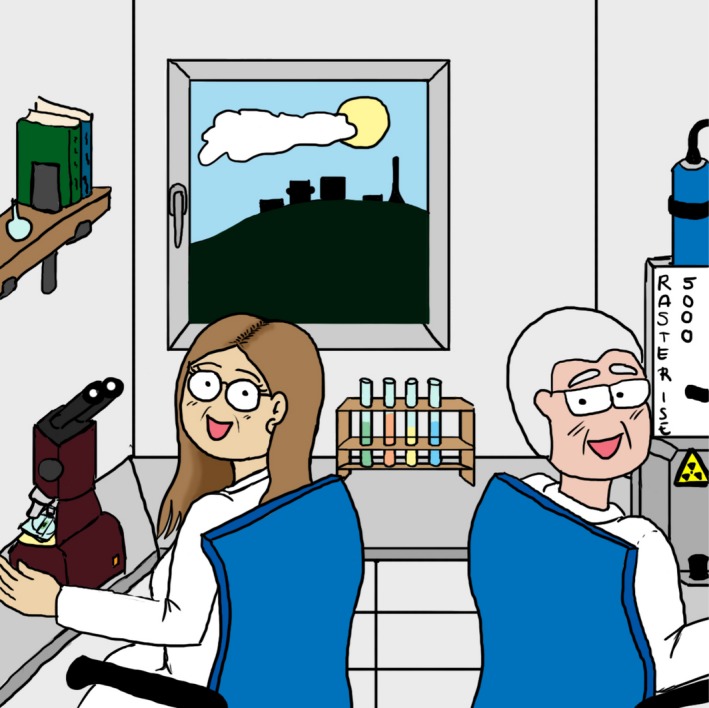

And indeed they were able to isolate two closely related bacteria that were abundant in slobber‐super dogs but undetectable in slobber‐poor dogs. When these bugs were given to slobber‐poor dogs in a commercial dog food, they caused the subjects to produce so much slobber that they left a trail wherever they went in the house – more or less like a snail‐slug trail, but rather more impressive. Unfortunately, one dog quickly ruined a rather expensive oriental rug in the house of its house‐proud owner, and another made a bit of a mess of the linen on the children's bed on which it slept. AND, crucially, both ruined the best shoes of the ladies of the households, so the trial was aborted because the dogs were suffering unnecessarily from the unhappiness of their usually happy owners, and all the screaming going on. Nevertheless, the new bacteria were unambiguously shown to play a major role in slobber production and were given the names *Slobbercopius paintballa* and *S. jettisona*, which they nicknamed *Slop* and *Sloj*.

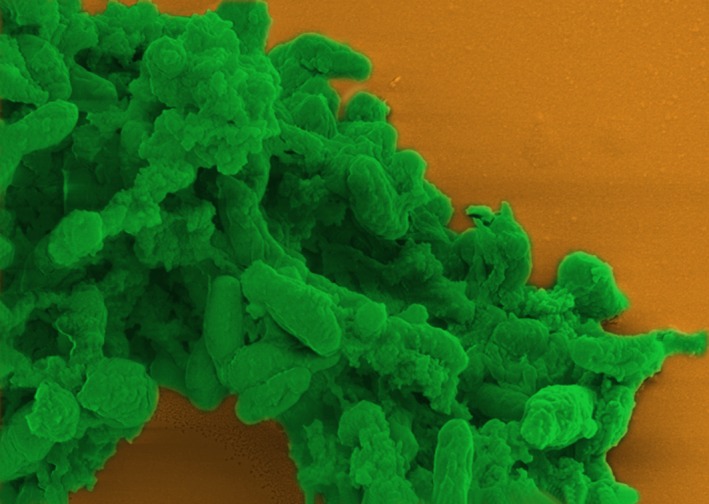

*Slop* 28

*Ms. Repor‐Tastory:* Oh, poor dogs!

*Dr. Noitall‐Most:* Quite! Anyway, initial studies to determine how *Slop* and *Sloj* induce slobber flow suggests that they produce both a hormone‐like compound they designated canine slobber‐inducing compound, or *CSIC*, that turns up production of saliva by the dog, and a new biosurfactant^29^ they designated soap‐like oral substance, or *SLOSH*, that acts like soap, turning all that extra saliva into a soapsud‐like slobber full of microbes growing in the buccal cavity.

*Ms. Repor‐Tastory:* Wow: so slobber is microbially‐triggered!

Ok, so finally, let me come to my traditional closing question: are there any applications of this part of the *GLOPS* investigation?

*Dr. Noitall‐Most:* Yes, of course, Abi! Firstly, there is the key issue of maintaining diversity of our microbiomes. While some folks are able to have pets and live next to the municipal waste treatment facility, many are not, so all the major food companies are developing so‐called microbiota diversity enhancement treatments, usually bug‐packed capsules that can be taken with a cup of cocoa, or a single malt and water, just before retiring. The capsule fillings are mostly spores of a wide range of microbes found in the environment.

The earliest to come to market will be based on microbes washed from crop plants, because regulatory approval is a simple process, since we already consume such bugs in our diet and the treatment does not represent anything fundamentally new. However, these microbe mixes are not exceptionally diverse, so other sources are being explored, including compost, but these will have to undergo rigorous safety testing before they will be authorised.

*Ms. Repor‐Tastory:* Oh: I am not sure I would want to swallow compost last thing at night…..

*Dr. Noitall‐Most:* Well: there is always the option of mucking out the stables……. But, seriously: I have heard on the grapevine that the public health department of one state that is rather wealthy and technologically advanced will soon carry out a limited trial involving the mass feeding of an aerosol loaded with a highly diverse but well characterised microbial flora into its metro system. Apparently, there has been a high level of uptake of an offer of lifelong free healthcare for regular metro‐users in exchange for regular detailed monitoring over a 10‐year period, so this trial will be exceptionally well documented and will show whether or not environmental engineering for microbial diversity is helpful for our health and, if so, in what ways. And….this trial without doubt marks the beginning of a new era of pro‐active environmental engineering interventions by public health agencies for microbiota delivery for different health objectives.

*Ms. Repor‐Tastory:* Gosh! But, then, such public health environmental engineering campaigns are not in principle different from the practice of adding iodide to table salt to eliminate iodine deficiency in the population,^30^ or of fluoride to table salt, drinking water and toothpaste, to reduce dental caries^30^ or, for that matter, distributing rabies vaccine in the countryside to eliminate rabies in foxes and the like.^30^ But live organisms in the metro? I have always thought that microbe transmission in subways was something to be avoided!

*Dr. Noitall‐Most:* Yes, Abi, you are right. But the thing is: while the effectiveness of microbe transmission in semi‐enclosed spaces is bad news for transmission of pathogens, it is good news for the transmission of healthy bugs. And, of course, one might be forgiven for considering the possibility that there may be additional, undeclared reasons for this trial, such as removal of the motivation to live all on top of one another near waste treatment facilities, etc., and the unnecessary and unsustainable rises in accommodation prices in such areas.

*Ms. Repor‐Tastory:* Oh, yes: it would be soooo nice to abandon the pig slurry and hound pack in favour of a quiet garden smelling of roses!

*Dr. Noitall‐Most:* Mind you: subways are not a particularly efficient means of distributing anything, because of the enormous dilution effect, the total volume:personal space volume ratio. So, if the practice of public health environmental engineering, or *PHEE*, really gets going, for example for annual mass immunization against prevalent flu strains, or to distribute vaccines against the usual childhood infections, it will more probably involve people walking through a sort of dosed air blade^31^ placed at head height, while taking a deep breath.

*Ms. Repor‐Tastory:* Gosh, how interesting: this is sort of *PHEE* would be equivalent to a sheep dip.^32^

*Dr. Noitall‐Most:* Errr, yes: thank you Abi!

But perhaps the most exciting application of these findings is being enthusiastically explored by the Lorenzo von Syntech High Security Institute for Artificial Life in Madrid, headed by the world‐renowned Professor Vic Torde, is the use of synthetic biology approaches to engineer an undisclosed super gut coloniser producing high levels of micronutrients we cannot make ourselves, starting with vitamin B12.^13^ The goal here is that such a bug, once introduced into our GI tract, would ensure that no‐one would suffer from micronutrient deficiencies any more.

*Ms. Repor‐Tastory:* Oh, this sounds like an even more promising development to allow me to abandon my vitamin pills in favour of a gym upgrade!

*Dr. Noitall‐Most:* Another application results from the finding that the *SLOSH* biosurfactant is exceptionally effective at removing wine stains from cotton and other fabrics, and hence is being developed as an upmarket bio‐stain remover – this will undoubtedly go like a bomb with organitarian wine enthusiasts. However, it turns out that production levels of *SLOSH* by *Slop* and *Sloj* grown in fermenters are so low that it cannot yet be commercialised.^33^ To get around the low production levels, Vic is also using synthetic biology approaches to design a new cell factory based on a genetically‐stripped down *Pseudomonas* KT2440 chassis^34^ that channels almost all of its carbon and energy to the manufacture of *SLOSH*. Interestingly, as viewers can see on the image now being shown,^35^ this strain produces *SLOSH* in the form of micropearls.

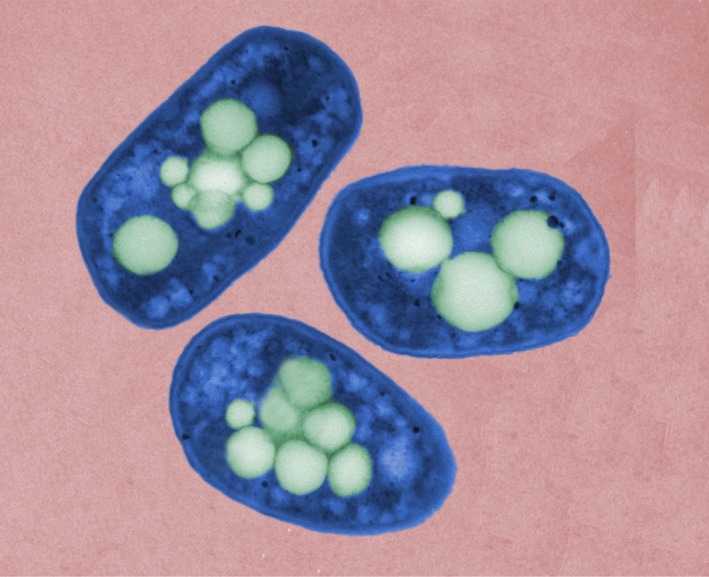

The researchers at LvS have given this form of the surfactant the name synthetic biology soap‐like oral substance micropearl stain remover, or *SYBSLOSMPSR (pronounced sib‐slos‐empeser),* although the company involved in its commercial exploitation has intimated that this name may not be outstandingly attractive for marketing purposes.

*Ms. Repor‐Tastory:* Gosh: they really do look like pearls! But how do the LvS researchers get the pearls out of the bacteria?

*Dr. Noitall‐Most:* Excellent question, Abi. In fact, this is a fundamental problem of some microbial biotechnology processes – the extraction of intracellular products – that has occupied researchers for some years now.^36^ But now, a promising new approach has been developed exploiting predatory bacteria, like *Bdellovibrio*, to lyse the cells and release their contents,^36^ including micropearls.

In fact, the KT2440 cell factory has worked out so amazingly well that Vic and his associates are now introducing into it biochemical pathways for the creation of new variants by modification of the *SLOSH* chemical skeleton. The first variant created has been shown to act as an emulsifier at ambient temperatures and to become irreversibly rigid at high temperatures, and moreover has a flavour of chocolate. Because a brief whipping of solutions containing this variant gives a mousse‐like product which becomes perfectly stabilised during cooking, Vic is exploring its use to create the perfect chocolate mousse.

Even more interestingly, he finds that the chemical tuning of a couple of parts of the *SLOSH* molecule yields all sorts of new flavours, reminiscent of raspberry, artichoke, fermented herring, etc., so believes that he will be able to discover entirely new flavours for the chefs of tomorrow.

*Ms. Repor‐Tastory:* Golly: that is absolutely fascinating. Well, viewers: on that culinary note, we end this edition of ‘Discoveries that Change our Lives'. We will be back next week with a follow‐up edition to reveal some more of the amazing findings of *GLOPS* and its experimental follow‐up.

++++++++

## Explanatory Notes for Online Version at www.theabsurdmicrobe.com

1 To view previous issues of *Microbiome Discoveries that Change our Lives*, go to http://www.theabsurdmicrobe.com/discoveries-oldstuff

2 Health Warning. This article is a figment of the authors' imagination; any serious contemplation may unnecessarily challenge the mental harmony of the reader.

3 Any possible resemblance of the fictional characters and organisations in this fable to a real person, organisation or enterprise is coincidental and unintentional.

4 Dedicated to pioneering researchers of the microbiome, synthetic microbiology, air microbiology, biosurfactants, to public health agencies everywhere for protecting us from all manner of health hazards, to Antoine Danchin for always making available his encyclopaedic knowledge, sharp insight and advice, and, as always, to the memory of Dan Koshland, Jr., father of Dr. Noitall (e.g. see Editorial: A simple proposal for health care rationing. *Science* 264: 1515), who in turn is the father of Dr. Anastasia Noitall‐Most, and David Jones, father of Daedalus, who invented Dreadco (see, e.g. https://www.newscientist.com/article/mg23531413-500-what-did-daedalus-talk-about-in-septembers-past/; https://www.improbable.com/2017/07/27/sad-news-daedalus-david-jones-is-gone/).

5
http://www.theabsurdmicrobe.com/discoveries/

6 Timmis, K., Jebok, F., Rohde, M and Molinari, G. (2019) Microbiome yarns: the global phenotype‐genotype survey. Episode I: all my worldly goods, including my microbiome, I thee endow. *Micro Biotechnol* **12**: 11–24; Timmis, K., Jebok, F., Rohde, M and Molinari, G. (2019) Microbiome yarns: the global phenotype‐genotype survey. Episode II: laryngeal microbiota and vocal phenotypes (*or diction and addiction*). *Micro Biotechnol* **12**: 191–199.

7 Kang, K, Ni, Y., Li, J., *et al*. (2018) The environmental exposures and inner‐ and intercity traffic flows of the metro system may contribute to the skin microbiome and resistome. Cell Rep *24*, 1190–1202; Herfst, S., Böhringer, M., Karo, B., *et al*. (2017) Drivers of airborne human‐to‐human pathogen transmission. *Curr Opin Virol* **22**: 22–29; Browne, H. P., Neville, B. A., Forster, S. C. and Lawley, T. D. (2017) Transmission of the gut microbiota: spreading of health. *Nat Rev Microbiol* **15**: 531–543; Maritz, J. M, Sullivan, S. A., Prill, R. J. *et al*. (2017) Filthy lucre: A metagenomic pilot study of microbes found on circulating currency in New York City. *PLoS One* **12**: e0175527.

8 Song, S. J., Lauber, C., Costello, E. K., Lozupone, C. A., Humphrey, G., Berg‐Lyons, D., *et al*. (2013). Cohabiting family members share microbiota with one another and with their dogs. *eLife*,** 2**, e00458. https://doi.org/10.7554/elife.00458; Jatzlauk, G., Bartel, S., Heine, H. and Kraus‐Etschmann, S. (2017) Influences of environmental bacteria and their metabolites on allergies, asthma, and host microbiota. *Allergy* 72: 1859–1867; Trinh, P., Zaneveld, J. R., Safranek, S. and Rabinowitz, P. M. (2018) One Health relationships between human, animal, and environmental microbiomes: a mini‐review. *Front Public Health*. **30:** 6: 235; Sharma, A. and Gilbert, J. A. (2018) Microbial exposure and human health. *Curr Opin Microbiol*. **44**: 79–87; Cardona, C., Lax, S., Larsen, P. *et al*. (2018) Environmental sources of bacteria differentially influence host‐associated microbial dynamics. *mSystems* **3**: e00052–18.

9 https://en.wikipedia.org/wiki/Particulates; Albrecht, A., Witzenberger, R., Bernzen, U. and Jäckel, U. (2007) Detection of airborne microbes in a composting facility by cultivation based and cultivation‐independent methods. *Ann Agric Environ Med*. **14**: 81–85; Gwang Pyo, K., Simmons III, O. D., Likirdopulos, C. A., *et al*. (2008) Investigation of bioaerosols released from swine farms using conventional and alternative waste treatment and management technologies *Environ. Sci. Technol*. 42: 8849–8857; Sánchez de la Campa, A., García‐Salamanca, A., Solano, J., de la Rosa, J. and Ramos, J. L. (2013) Chemical and microbiological characterization of atmospheric particulate matter during an intense African dust event in Southern Spain. *Environ Sci Technol*. 47: 3630–3638; O'Brien, K. M., Chimenti, M. S., Farnell, M. *et al*. (2016) High throughput genomic sequencing of bioaerosols in broiler chicken production facilities. *Microb Biotechnol* **9**: 782–791; Joung, Y. S., Ge, Z. and Buie, C. R. (2017) Bioaerosol generation by raindrops on soil. *Nat Commun* **8**: 14668; Cáliz, J., Triadó‐Margarit, X., Camarero, L. and Casamayor, E. O. (2018) A long‐term survey unveils strong seasonal patterns in the airborne microbiome coupled to general and regional atmospheric circulations. *Proc Natl Acad Sci USA* **115**: 12229–12234; Park, J., Li, P. F., Ichijo, T., Nasu, M. and Yamaguchi, N. (2018) Effects of Asian dust events on atmospheric bacterial communities at different distances downwind of the source region*. J Environ Sci (China)* **72**: 133–139; Wang, Y., Lan, H., Li, L. *et al*. (2018) Chemicals and microbes in bioaerosols from reaction tanks of six wastewater treatment plants: survival factors, generation sources, and mechanisms. *Sci Rep* **8**: 9362; Yang, W., Guo, M., Liu, G., *et al*. (2018) Detection and analysis of fine particulate matter and microbial aerosol in chicken houses in Shandong Province, China*. Poult Sci* **97**: 995–1005; Evans, S. E., Dueker, M. E^.^, Logan, J. R. and Weathers, K. C. (2019) The biology of fog: results from coastal Maine and Namib Desert reveal common drivers of fog microbial composition. *Sci Total Environ* **647**: 1547–1556.

10 Obersteiner, A., Gilles, S., Frank, U., *et al*. (2016) Pollen‐associated microbiome correlates with pollution parameters and the allergenicity of pollen. *PLoS One* **11**: e0149545; Ambika Manirajan, B., Ratering, S., Rusch, V. *et al*. (2016) Bacterial microbiota associated with flower pollen is influenced by pollination type, and shows a high degree of diversity and species‐specificity. *Environ Microbiol* **18**: 5161–5174; Zasloff, M. (2017) Pollen has a microbiome: implications for plant reproduction, insect pollination and human allergies. *Environ Microbiol* **19:** 1–2; Manirajan, B. A., Maisinger, C., Ratering, S., *et al*. (2018) Diversity, specificity, co‐occurrence and hub taxa of the bacterial‐fungal pollen microbiome. *FEMS Microbiol Ecol*. **94**. https://doi.org/10.1093/femsec/fiy112.

11 No, sorry, this is bit of a stretch of artistic licence: pig slurry is not generally considered to be particularly beneficial to country air quality and, for large scale hog farming operations, may constitute a significant source of air, soil and water pollution: https://www.theguardian.com/environment/2018/may/24/pig-farm-agriculture-its-wrong-to-stink-up-other-peoples-lives-fighting-the-manure-lagoons-of-north-carolina; https://modernfarmer.com/2014/01/stink-wars-foul-wind-wafts-farm-problem/; http://ec.europa.eu/environment/air/pdf/TSAP-AGRI-20121129_v21.pdf;

12 Kearney, S. M., Gibbons, S. M., Poyet, M., *et al*. 2018. Endospores and other lysis‐resistant bacteria comprise a widely shared core community within the human microbiota. *ISME J*. doi.org/10.1038/s41396‐018‐0192‐z. The gorgeous image shows cells of *Bacillus subtilis* strain 1186, which produces secondary metabolites having antimicrobial activities, lysing and releasing spores.

13 Danchin, A. and Braham, S. (2017) Coenzyme B12 synthesis as a baseline to study metabolite contribution of animal microbiota. *Microbio Biotech* **10**: 688–701; Danchin, A. (2018) Bacteria in the ageing gut: did the taming of the fire promote a long human lifespan? *Environ Microbiol* 20: 1966–1987.

14
https://www.youtube.com/watch?v=rgphaHmAC_A

15 Diard, M. and Hardt, W. D. (2017) Evolution of bacterial virulence. FEMS Microbiol Rev 41: 679–697; Merrikh, C. N. and Merrikh, H. (2018) Gene inversion potentiates bacterial evolvability and virulence. Nat Commun 9: 4662; Williams, P. D. and Kamel, S. J. (2018) The evolution of pathogen virulence: Effects of transitions between host types. J Theor Biol 438: 1–8.

16 Timmis, K., Jebok, F., Molinari, G., Rohde, M. and Lahti L. (2018) Microbiome Yarns: human biome reproduction, evolution and visual acuity. Microb Biotechnol 11: 149–159; for another example of floor/near floor microbial bounties differing from bulk air bounties, see also Hyytiäinen, H. K., Jayaprakash, B., Kirjavainen, P. V., *et al*. (2018) Crawling‐induced floor dust resuspension affects the microbiota of the infant breathing zone. *Microbiome* **6**: 25; https://www.telegraph.co.uk/travel/maps-and-graphics/the-tallest-and-shortest-countries-in-the-world/.

17 This is a scanning electron micrograph of dust, unfortunately not the floor variety assiduously vacuumed up by infants – the subject under discussion – but from a somewhat neglected window ledge which, however, shows beautifully diverse biological entities among rather amorphous particulate grunge.

18 Finlay, B. B, and Arrieta, M.‐C. (2016) Let them eat dirt: how microbes can make your child healthier. Windmill Books (see also section entitled *Bring on the slobberfest*); Gilbert, J. A., Knight, R. and Blakeslee, S. (2017) Dirt is Good. St Martin's Press (https://us.macmillan.com/books/9781250132604).

19 Kelley, S. T., Theisen, U., L. T., St. Amand, A. and Pace, N. R. (2004) Molecular Analysis of Shower Curtain Biofilm Microbes**.** Appl Environ Microbiol 70: 4187–4192; Mukherjee, N., Dowd, S. E., Wise, A., *et al*. (2014) Diversity of Bacterial Communities of Fitness Center Surfaces in a U.S. Metropolitan Area. *Int J Environ Res Pub Health* **11**: 12544–12561; Bonifait, L., Marchand, G., Veillette, M., *et al*. (2017) Workers' exposure to bioaerosols from three different types of composting facilities. *J Occup Environ Hyg* **14**: 815–822; Khan, H. A., Baig, F. K. and Mehboob, R. (2017) Nosocomial infections: Epidemiology, prevention, control and surveillance. *Asian Pacific J Trop Biomed* 7: 478–482; Price, L. B., Hungate, B. A., Koch, B. J., Davis, G. S. and Liu, C. M. (2017) Colonizing opportunistic pathogens (COPs): The beasts in all of us. *PLOS Pathogen* **13**: e1006369.

20 Zimmer, C. (2010) Yet‐another‐genome syndrome. *Discover Mag* http://blogs.discovermagazine.com/loom/2010/04/02/yet-another-genome-syndrome/#.XBbWMc1OlhE

21 Schmid‐Hempel, P. and Frank, S. A. (2007) Pathogenesis, virulence, and infective dose. *PLoS Pathog* 3: e147; Kothary, M. H. and Babu, U. S. (2007) Infective dose of foodborne pathogens in volunteers: a review. J Food Safety 21: https://doi.org/10.1111/j.1745-4565.2001.tb00307.x

22 Miller, M. B. and Bassler, B. L. (2001) Quorum sensing in bacteria. *Annu Rev Microbiol* **55**: 165–199; Sturm, A., Heinemann, M., Arnoldini, M., *et al*. (2011) The cost of virulence: retarded growth of *Salmonella Typhimurium* cells expressing type III secretion system 1. *PLoS Pathog* **7**: e1002143; Kitamoto, S., Nagao‐Kitamoto, H., Kuffa, P. and Kamada, N. (2016) Regulation of virulence: the rise and fall of gastrointestinal pathogens. *J Gastroenterol* **51**: 195–205; Personnic, N., Striednig, B. and Hilbi, H. (2018) *Legionella* quorum sensing and its role in pathogen‐host interactions. *Curr Opin Microbiol* **41**: 20–35.

23
https://en.wikipedia.org/wiki/Strange_Case_of_Dr_Jekyll_and_Mr_Hyde; https://www.gutenberg.org/files/43/43-h/43-h.htm

24
https://www.cdc.gov/legionella/about/causes-transmission.html; Huehn, S., Eichhorn, C., Urmersbach, S., *et al*. (2014) Pathogenic vibrios in environmental, seafood and clinical sources in Germany. *Int J Med Microbiol* **304**: 843–850; Wynwood, S. J., Graham, G. C., Weier, S. L., *et al*. Leptospirosis from water sources. *Pathog Glob Health* **108**: 334–338.

25 Timmis, K., Jebok, F., Rigat, F. M., de Vos, W. and Timmis, J. K. (2017) Microbiome yarns: microbiomology of curly and straight hair. Microb Biotechnol 10: 231–237.

26
*SLIND* (slobber index) the amount of foam produced and accumulating in and around the mouth, prior to distribution by centrifugal head action, shaking or dripping. It ranges from 1 to 100, with 1 representing essentially no slobber (essentially the dog is moribund) and 100, representing serious distribution of 10^13^ microbes per day, typically in 1litre of slobber. The formula derived by Professor Fidget is:

$$\begin{array}{cc} & SLIND=\Psi^{-1}\left(\theta \right)\left\{\frac{\left(W\left(W-5\right)F_{o}T_{o}T\left[\left(A-2\right)\left(A-3\right)+1\right]\right)}{S^{P}F_{t}Eeek_{c}Eeek_{o}Eeek_{p}f\left(D\right)\left(2-B\right)N}\right\}+c\left(\theta \right) \end{array}$$

where *W *= dog weight, a variable with values of 0 (= < 2kg), 1 (= 2–5 kg), 2 (= 5–10 kg), 3 (= 10–20 kg), 4 (= 20–40 kg), 5 (= 40–60 kg) and 6 (= > 60 kg), *N* = neck muscular strength, approximated by the number of seconds it takes to extract a tennis ball from the dog's mouth, *T*_*o*_ = tongue length in cm, *T* = tail factor: weight x length of tail (provides body stability to, and increases the thrust of, the neck movements involved in slobber scattering), *A* = age of the dog, with values of 1–4, corresponding to puppy (1), teenage (2), adult (3) and pensioner (4), with maximal activity for 2 and 3, and reduced activity for 1 and 4, *S* = frequency of sneezes precipitating slobber distribution, with values of 1 (usually no sneezes) or 2 (usually multiple sneezes), *F*_*t*_ = average number of faecal treats allowed by mum or dad per walkie, approximated by the number of social media messages received per hour, with values of 0 for 0–2 messages, 1 for 3–5 and 2 for 6+,*P* = frequency of neck flicks provoked by fleas having their blood snacks: equates with external parasite load, which tends to increase both scratching and slobber distribution, and is approximated by the number of fleas collected by one pass of the flea comb over the back of the neck, with values of 0 for <2 fleas in evidence, 1 for 3–5, and 2 for 6+,*Eeek*_*c*_ **= level of excitement, averaged over the day, caused by excitable children stepping on tail, with a fixed variable of 5.6 for 0–5 events, *Eeek*_*o*_ **= level of excitement caused by becoming oestrous (female) or by proximity to an oestrous female (male), with values of either 0 (no oestrous female influence) or 27, *Eeek*_*p*_ = frequency of chase‐able animals in the environment, such as a poodle next door, a cat, a fox (especially hunting dogs), a fixed variable of 3.77, *D* = dehydration factor = frequency of water bowl drying out because owner forgets to fill – approximated by the number of units of alcohol consumed per day, where a 1–3 unit consumption has a value of 1, 4–6 units has a value of −12, 6–10 units has a value of −16, and 10–20 is lethal (for both the owner and the dog), and the *SLIND* value necessarily becomes 1, *F*_*o*_ = frustration term for the lack of responsiveness of owners to requests for walkies to empty bladder, a variable with a fixed value of √7/19π*B* = barking frequency, with values of 2 for 2 hours of barking and ‐2 for longer periods. A *SLIND* value obtained is then used to calculate the *α diversity* allergy susceptibility of a family member, using the following relationship:

$$SLIND∼N\left(\right.\alpha ,\xi |\left[\alpha_{O}+I_{m}\alpha_{C},\xi_{m}\alpha_{C}\right],\sigma^{2}I$$

The diversity (*α*) and susceptibility index (*ξ*) have a joint distribution, described by multivariate normal. Diversity is obtained by summing up population baseline diversity *α*_0_, and then canine members diversity *α*_c_ multiplied by immigration rate *I*_m_ between the canine and family member m. Then some noise is added so that the diversity in family member m is only approximately sum of baseline diversity and canine diversity. Susceptibility index is calculated by multiplying the personal susceptibility of family member m with microbiome diversity in the canine.

27 Timmis, K., Jebok, F., Molinari, G., Rohde, M. and Timmis, J. K. 2017. Microbiome yarns: microbiome basis of memory. *Microbiol Biotechnol* **10:** 1283–1292.

28 No, sorry: this is not *Slobbercopius paintballa*, but anyway, so intricately beautiful and menacing, that we decided to take full advantage of artistic licence and use it for illustrative purposes. Without decoration, it is designated 2D9, was isolated by Ken Timmis, Joan Timmis and Balbina Nogales from the phylosphere of some beautiful plant or other on the campus of the University of the Balearic Isles, perhaps on a walkie route, while KT was a short‐term junior postdoc of Balbina Nogales, to whom he expresses infinite gratitude for her wonderful mentoring, tolerance, patience and determined refusal to be driven insane in the face of apocalyptical chaos, and to the UIB for a grant supporting his stay in Palma. Back in Braunschweig, Gabriella Molinari and Manfred Rohde revealed the beautiful morphology of 2D9 and discovered that it is distantly related to *Quadrisphaera granulorum* (Maszenan, A.M., Tay, J.H., Schumann, P., Jiang, H.L. and Tay, S.T. (2005) *Quadrisphaera granulorum* gen. nov., sp. nov., a Gram‐positive polyphosphate‐accumulating coccus in tetrads or aggregates isolated from aerobic granules. *Int J Syst Evol Microbiol* **55**: 1771–1777.) which is, however, distinctly boring in appearance.

29 Yakimov, M., Timmis, K. N., Wray, V. and Fredrickson, H. L. (1995) Characterization of a new lipopeptide surfactant produced by thermotolerant and halotolerant subsurface *Bacillus licheniformis* strain BAS50. *Appl Environm Microbiol* **61**:1706–1713; Yakimov, M. M., Fredrickson, H. L. and Timmis, K. N. (1996) Effect of heterogeneity of hydrophobic moieties on surface activity of lichenysin A, a lipopeptide biosurfactant from *Bacillus licheniformis* BAS50. *Biotech App Biochem* **23**: 13–18; Desai, J. D. and Banat, I. M. (1997) Microbial production of surfactants and their commercial potential. *Microbiol Mol Biol Rev* **61**: 47–64; Yakimov, M. M., Golyshin, P. N., Lang, S., *et al*. (1998) *Alcanivorax borkumensis* gen. nov. sp. nov., a new, hydrocarbon‐degrading and surfactant‐producing marine bacterium. *Int J Syst Bact* **48**: 339–348; Gudina, E. J., Teixeira, J. A. and Rodrigues, L. R. (2016) Biosurfactants produced by marine organisms with therapeutic applications. Mar Drugs 14: https://doi.org/10.3390/md14020038

30 World Health Organization; UNICEF; ICCIDD. (2007) Assessment of the iodine deficiency disorders and monitoring their elimination. World Health Organization Press; Geneva, Switzerland; Prete, A., Paragliola, R. M. and Corsello, S. M. (2015) Iodine supplementation: usage ‘with a grain of salt' *Int J Endocrinol* **2015**: 312305; Marthaler, T. M. and Petersen, P. E. (2005) Salt fluoridation – an alternative in automatic prevention of dental caries. *Int Dental J* **55**: 351–358; Müller, T. F., Schröder, R., Wysocki, P., *et al*. (2015) Spatio‐temporal use of oral rabies vaccines in fox rabies elimination programmes in Europe. *PLoS Negl Trop Dis* **9**: e0003953.

31
https://en.wikipedia.org/wiki/Dyson_Airblade

32 https://en.wikipedia.org/wiki/Sheep_dip; NB: **NOT** https://www.urbandictionary.com/define.php?term=sheep%20dipped, **NOR** https://www.opswat.com/blog/what-does-sheep-dip-mean-cyber-security, **NOR** https://www.masterofmalt.com/whiskies/sheep-dip/sheep-dip-whisky/, **NOR** http://www.sheep-dip.de

33 see also: Yakimov, M. M., L. Giuliano, K. N. Timmis and P. N. Golyshin. 2000. Recombinant acylheptapeptide lychenysin: high level of production by *Bacillus subtilis* cells. *J Mol Microbiol. Biotech* 2: 217–224.

34 Bagdasarian, M., Lurz, R., Rückert, B., *et al*. (1981) Specific‐purpose plasmid cloning vectors. II. Broad host range, high copy number, RSF1010‐derived vectors, and a host‐vector system for gene cloning in *Pseudomonas*. *Gene* **16**: 237–247; Timmis, K. N. (2002) *Pseudomonas putida:* a cosmopolitan opportunist *par excellence. Environm Microbiol* **4:** 779–781; Nelson, K. E., Weinel, C., Paulsen, I. T., *et al*. (2002) Complete genome sequence and comparative analysis of the metabolically versatile *Pseudomonas putida* KT2440. *Environm Microbiol* **4:** 799–808; Regenhardt, D., Heuer, H., Heim, S., *et al*. (2002) Pedigree and taxonomic credentials of *Pseudomonas putida* strain KT2440. *Environm Microbiol* **4**: 912–915; Belda, E., van Heck, R. G. A., Lopez‐Sanchez, M. A., *et al*. (2016) The revisited genome of *Pseudomonas putida* KT2440 enlightens its value as a robust metabolic *chassis*. *Environ Microbio* **18**: 3403–3424; Nikel, P.I. and de Lorenzo, V. (2018) *Pseudomonas putida* as a functional *chassis* for industrial biocatalysis: from native biochemistry to *trans*‐metabolism. *Metab Eng* **50**: 142–155

35 Erm, no, sorry: while this is a gorgeous depiction of the renowned cell factory bacterium, *Pseudomonas* strain KT2440 (the species name is in a bit of state of flux at the moment), some artistic license has been exercised and the inclusions are not *SYBSLOSMPSR* micropearls, but rather granules of polyhydroxyalkanoate (see Poblete‐Castro, I., Binger, D., Oehlert, R., and Rohde, M. (2014) Comparison of mcl‐poly (3‐hydroxyalkanoates) synthesis by different *Pseudomonas putida* strains from crude glycerol: citrate accumulates at high titer under PHA‐producing conditions. *BMC biotechnol* 14, 962 https://doi.org/10.1186/s12896-014-0110-z)

36 Harrison, S. T. L. (1991) Bacterial cell disruption: A key unit operation in the recovery of intracellular products. *Biotechnol Adv* **9:** 217–240; Martinez, V., Herencias, C., Jurkevitch, E. and Prieto, A. (2016) Engineering a predatory bacterium as a proficient killer agent for intracellular bioproducts recovery: the case of the polyhydroxyalkanoates. *Sci. Rep* **6**: 24381.

